# Electromyographic parameters for treatment of pelvic floor disorders in pregnant and postpartum women: A review protocol

**DOI:** 10.1371/journal.pone.0309822

**Published:** 2024-11-04

**Authors:** Alethéa Cury Rabelo Leitão, Silvia Oliveira Ribeiro Lira, Elizabel de Souza Ramalho Viana

**Affiliations:** 1 Department of Physical Therapy, Universidade Federal do Rio Grande do Norte, Natal, Rio Grande do Norte, Brazil; 2 Federal University of Rio Grande do Norte, Natal, Rio Grande do Norte, Brazil; 3 Faculdade de Ciências da Saúde do Trairi (FACISA/UFRN), Santa Cruz, Rio Grande do Norte, Brazil; Iran University of Medical Sciences, ISLAMIC REPUBLIC OF IRAN

## Abstract

Electromyography is a widely used instrument in clinical practice to evaluate and treat pelvic floor disorders in pregnant and postpartum women. The objective of this study is to analyze the scientific evidence on the electromyography parameters used for treatment of pelvic floor disorders in pregnant women in any gestational week and postpartum women up to 12 months after delivery. A systematic review of randomized controlled experimental studies (clinical trials) and quasi-experimental studies in English, Portuguese or Spanish, which used electromyography as an intervention for treatment of pelvic floor disorders in pregnant or postpartum women up to 12 months after delivery will be performed in online databases (Scopus, Medline, Pedro, Scielo and Pubmed),. Risk of bias assessment will be performed using Cochrane group tools. The Rob 2.0 tool will be used for experimental studies and the Robins-I tool for non-experimental studies. The protocol was registered in PROSPERO (no.433510). The quality of the evidence will be analyzed using the GRADE System Methodological Guide and the systematic review structure will be performed according to the Preferred Reporting Items for Systematic Reviews and Meta-Analyses (PRISMA) guidelines.

## Introduction

### Description of the condition

It is consensus that the risk factors for developing pelvic floor disorders in women are related to the pregnancy and delivery period [1]. The literature shows that 50% of women lose some support functionality of the pelvic floor muscles (PFM) due to childbirth, and these injuries increase by an average of 20% in women who had vaginal delivery [2].

The pelvic floor of these women will be overloaded during pregnancy due to enlargement of the gravid uterus from anatomical and physiological changes [3]. Deficiency in the pelvic floor muscles (PFM) can lead to developing stress urinary incontinence (SUI), fecal incontinence (FI), and pelvic organ prolapse (POP) [3, 4]. These pelvic floor dysfunctions (PFD) negatively impact the quality of life in women, creating a significant social problem [4].

A recent study indicates that women who had vaginal deliveries exhibited greater pelvic floor muscle weakness compared to those who had cesarean sections. The proposed treatment utilized various electromyographic parameters to rehabilitate pelvic floor muscle dysfunctions caused by muscle fiber rupture during childbirth [5].

Pelvic floor muscle training (PFMT) is the first-line treatment for dysfunctions of this musculature [6]. This treatment is based on increasing strength, endurance, maintaining muscle contraction for a long period of time, muscle coordination, adherence to and motivation for the training program [7, 8].

The literature includes proposed protocols that supplement pelvic floor muscle training (PFMT) with surface electromyography (sEMG) for strengthening the pelvic floor muscles in treatment of stress urinary incontinence (SUI) in pregnant women [9, 10].

It is necessary to emphasize the importance of defining the electromyographic parameters used in treatment of pelvic floor muscle dysfunctions (PFMD). Standardizing these parameters will enable professionals to reproduce and validate results in scientific studies, thereby contributing to develop evidence-based therapeutic plans. Electromyography can be an adjunct to pelvic floor muscle training for treatment of urinary disorders in pregnant and postpartum women.

### Description of the intervention

Surface electromyography (sEMG) is a therapeutic modality applied to treat pelvic floor muscle (PFM) dysfunctions [1, 11]. sEMG is used to capture and read the myoelectric activity of the PFM during the treatment of pelvic floor dysfunctions [12], providing visual or auditory biofeedback (EMG-BF).

A 2021 meta-analysis with more than four thousand women found that the use of electromyography combined with conservative treatment of the pelvic floor muscles has better results than the isolated treatment [1]. In a meta-analysis of 11 studies, Nunes *et al*. (2019) [13] concluded that PFMT combined with sEMG offers therapeutic benefits over other interventions in treatment of female SUI [9]. Additionally, a systematic review by López-Liria *et al*. (2019) [14] analyzed the efficacy of different techniques in treatment of female SUI. The findings showed that sEMG, when combined with PFMT, is more effective in treatment of pelvic floor muscle dysfunctions (PFD) compared to other techniques analyzed. This effectiveness is attributed to the observed increase in muscle contraction.

There are several methods for evaluating and treatment of the functionality of the pelvic floor muscles, such as: digital palpation, manometry, ultrasound, electromyography and magnetic resonance imaging [6, 15].

sEMG provides important data on baseline tone, type of contraction, signal amplitude, changes in signal spectrum, amplitude variability, and recovery time of the pelvic floor muscles (PFM) [11]. These data are essential for describing muscle functionality during maximal or isometric contraction exercises and muscle behavior during rest [16].

### How the intervention will work

sEMG is an easily applicable, versatile, and comprehensible tool [3]. Clinical studies have demonstrated that conservative training with sEMG enables professionals and patients to accurately and objectively observe the contraction and relaxation of pelvic floor muscles (PFM) [11]. This facilitates neuromuscular learning and allows for more effective rehabilitation [8].

Electromyography can be used alone or in combination with conservative treatment. It enables indicating the activity of the pelvic floor muscles in relation to rest during contraction and during relaxation [13, 17].

Clinical studies have evaluated the bioelectric activity of this muscle group using electromyography [18]. This resource has been used as an adjunct to conservative training and enables the physiotherapist to correctly and objectively observe the contraction and relaxation of the pelvic floor muscles. Therefore, it facilitates neuromuscular learning and more assertively performs rehabilitation [13, 17].

More recent reviews which analyzed the effectiveness of conservative treatment with and without electromyography in relation to pelvic floor strength, urinary incontinence score and quality of sexual life excluded pregnant and postpartum women from the intervention groups to analyze these effects, leaving a gap in the literature for this population [14, 19, 20].

Knowledge about data on frequency, intensity and type of muscle contraction can more assertively determine goals and therapeutic conduct. The electromyographic parameters of the pelvic floor muscles guide the health professional in choosing the best strategies for the successful treatment of PFM disorders in pregnant and postpartum women. However, due to the scarcity of studies in this population, a systematic investigation of the literature on the conducts carried out and their effects so far is necessary.

### Why is this review important?

It is important to have a better understanding of the electromyography parameters most used in treatment of pelvic floor muscle disorders in pregnant and postpartum women. Identifying electromyography data can be a reference for elaborating pelvic floor rehabilitation procedures, such as data used for muscle strengthening or endurance training that improve vaginal occlusion in cases of stress urinary incontinence (SUI), and thereby contribute to prevent and treat dysfunctions in pelvic floor muscles. Furthermore, previous systematic reviews have not analyzed the most appropriate therapy for this population when using EMG alone or in combination in this population.

Therefore, this study aims to analyze the scientific evidence about electromyographic parameters in treatment of pelvic floor dysfunctions in pregnant and postpartum women.

## Materials and methods

### Protocol and guidelines

This review will be conducted following the Preferred Reporting Items for Systematic Reviews and Meta-Analyses (PRISMA) guidelines [21]. The protocol was registered with PROSPERO (no.433510) and will be conducted between August 2023 and October 2024.

### Type of studies

The review will include randomized controlled experimental studies (RCTs) and non-experimental studies (nRCTs), in English, Portuguese or Spanish, using sEMG in pregnant or postpartum women for treatment of pelvic floor dysfunctions (IUE) which analyzed baseline tone, contraction and relaxation capacity.

### Type of participants

Studies will include pregnant women (at any gestational week) and postpartum women (up to 12 months postpartum) with pelvic floor dysfunction (PFD) who were treated with EMG.

### Type of interventions

In addition, studies which used electromyography as an instrument, in isolation or in combination, for evaluating or treatment of of pelvic floor muscle dysfunctions that describe the parameters used in the therapy of these women will also be included.

### Type of outcome measures

#### Primary outcomes

1. Baseline pelvic floor muscle tone
2. Maximum voluntary contraction of the pelvic floor muscles
3. Sustained contraction of the pelvic floor muscles
4. Functionality of the pelvic floor muscles (contraction-relaxation coordination capacity)
5. Functionality of the pelvic floor muscles (ability for rapid contractions)

#### Secondary outcomes

6. Types of electromyographic equipment
7. Patient positioning during the intervention
8. Limitations of the chosen therapy
9. Types of Female Pelvic Floor Dysfunction More
10. Adverse events

#### Electronic database

1. Cochrane Central Register of Controlled Trials (CENTRAL)
2. MEDLINE (Pubmed)
3. Banco de Dados de Evidências em Fisioterapia (PEDro)
4. Scopus
5. Web of Science
6. Scielo
7. US National Institutes of Health, Register of Continuous Trials, ClinicalTrials.gov (www.clinictrials.gov);

*Literature search strategies*. The strategy on the terms used in each database can be found in S1 Appendix.

#### Searching other resources

Reference checking of primary studies will be done manually and review articles will be added to the references.

### Selection of studies

#### Data collection and analysis

For each search strategy, two reviewers will independently (ACRL and S) evaluate the studies from the databases in the order: title, abstract and full reading. Eligible studies will be read in full and data extracted for inclusion. The exclusion reasons for the studies will also be analyzed one by one. Disagreements regarding articles will be resolved by a third author by casting a vote (ESRV).

Duplicate studies will be identified and excluded. Studies involving men, children or non-pregnant women will also be excluded from the eligibility process and detailed in the Guideline Prisma flowchart.

#### Data extraction and management

The authors will extract the characteristics below from the included studies based on the PICO acronym [22]. The PICO acronym stands for Patient, Intervention, Comparison, and Outcome; these four components are fundamental elements in evidence-based practice for formulating research questions and conducting literature searches (Table 1)

1. Participants (P): *Inclusion criteria*. Pregnant women in any gestational phase or postpartum women up to 12 months after delivery, postpartum time, mean age, gestational week, floor dysfunction for treatment, sample inclusion and exclusion criteria, and sample description. *Exclusion criteria*. Women with neurological disease, urogenital tract infections.
2. Intervention (I): type of electromyographic equipment, types of electrodes, type of comparator equipment (if any), use of combined therapy (if any), intervention time, electromyographic parameters used, description of alternative interventions (placebo, no intervention or other intervention).
3. Comparator (C): different techniques or absence of intervention
4. Results (O): primary and secondary studies that evaluated electromyographic parameters for treatment of pelvic floor muscle dysfunctions.
5. Notes: authors, year of publication, funding of studies, and notable conflicts of interest among authors, study design, session time, follow-up time, study location, patient positioning, PFM functionality assessment method, treatment method.

**Table 1:** Study characteristics related to the number of participants, inclusion, and exclusion criteria.

**Author/ Year**	**Number of participants**	**Inclusion criteria**	**Exclusion criteria**

Two reviewers (ACRL and SORL) will perform the initial data extraction from the included studies after reading the full text and within the inclusion criteria. A “Summary of included studies” table will be created, informing the total number of studies. In case of disagreements in data extraction, a meeting will first be held for consensus on the extraction, and in the persistence of doubt, a third author will decide by casting a vote (ERSV). The review author (ACRL) will transfer the summarized data to the Systematic Reviews management program (RevMan 2014) in order to generate the study report and analysis of heterogeneity and the possibility of meta-analysis of the data.

In case of lack of important data to perform the analysis, the author (ACRL) will contact the authors to provide details of the study in question. A professional fluent in the English language or Google Translator will assist in the translation of other published languages in case of doubts. The main results will be carefully reanalyzed by the study authors after translation.

#### Risk of bias assessment in the included studies

Two independent authors (ACRL and SORL) will analyze the risk of bias of experimental studies using the Rob 2.0 tool and the Robins-I tool for non-experimental studies. Disagreements will be resolved by consensus or involving a third review author (ESRV).

The assessment of bias risk in experimental and quasi-experimental studies will be conducted according to the Cochrane Handbook of Intervention Systematic Reviews [23]. The selection of these study designs was based on the rationale that they provide therapeutic intervention data for a specific sample. For this research, the Rob 2.0 tools will be use for experimental studies, and Robins-I for non-experimental studies.

The Rob 2.0 tool is structured in five domains that have “signaling questions” with the possibility of answers in: “yes”, “probably yes”, “probably not”, “no”, “no information’” and “not applicable”. Definitive “yes” and “no” answers often indicate that robust evidence is available. The “not applicable” option is only available for questions with a non-mandatory answer. The final score of the responses determines the risk of bias for each domain: “high risk of bias”, “low risk of bias” or “unclear” [24].

The ROBINS-I tool evaluates seven domains of bias, classified by: low risk of bias, moderate risk of bias, severe risk of bias, critical risk of bias or no information. The result for the final analysis of each component of the domain is based on the answers to the guiding questions and tables that support the judgment of bias in each domain [25].

Other bias: the ROBINS-I tool also allows for ranking the overall risk of bias, which receives the least favorable ranking among the assessed risks for the assessment tool’s domains.

#### Evidence quality assessment

The evidence quality will be analyzed using the GRADE tool [26]. The structure of the systematic review will follow the recommendation of the Preferred Reporting Items of Systematic Reviews and Meta-Analyses (PRISMA) guidelines [21] and the protocol is registered in the PROSPERO database [27], the international prospective register of systematic reviews in health and social care (www.crd.york.ac.uk/prospero).

#### Assessment of bias during the systematic review

The review must be conducted according to this protocol and any adjustments can be justified in the “Difference between protocol and review” tab in the systematic review session.

#### Effect treatment measures

Dichotomous data: the odds ratios (ORs) and their associated 95% confidence intervals (CIs) will be used to determine the value of dichotomous data.

Continuous data: will be evaluated using standardized mean differences (STDs) and their corresponding 95% CIs using the Mantel-Haenzel method.

In case of a difference between means, the standard mean can be used for studies that analyzed the same result using different methods. P-values less than 0.05 will be considered statistically significant in all cases.

#### Issues related to a single analysis

Data analyzed in the study may be analyzed using a single analysis on the outcomes found. Meta-analysis may be used for randomized studies only if the data are justified for doing so.

#### Lost data

The authors of the present study will be able to contact the authors of the articles listed for data analysis in order to resolve doubts or request numerical data that were not made explicit in the body of the text or which were not found in the Register of Continuous Tests, ClinicalTrials.gov (www.clinictrials.gov). There are cases where only the abstract of the study contains the information, however, in the course of the text it does not. The failure of contacts and loss of entered data cause serious bias and this will be considered in the GRADE system.

### Evaluation of heterogeneity

If great heterogeneity is identified between the studies, the possible causes of the data discrepancy can be evaluated using specific sub-groups for analysis. If it is not possible to analyze the subgroup, the qualitative analysis will be summarized and presented in tables. In this case, the I^2^ statistic and the p-value obtained from the Cochrane’s Q test can be used.

### Evaluation of the risk of bias

The funnel chart can investigate reported biases. Symmetry of the funnel plot can be visually evaluated and explored. The analysis will be performed using the Egger’s test.

### Synthesis of the data

A random effects-model and sensitivity analysis with a fixed model can be used for subgroup analysis and data heterogeneity. It is suggested to follow the results below for subgroup analysis:

1. Electromyographic parameters for the treatment of stress urinary incontinence in pregnancy and postpartum women.
2. Limitations of the chosen therapy

The following results will be used in subgroup analyzes:

1. Functionality of the pelvic floor muscles
2. Most common types of female pelvic floor dysfunction
3. Adverse events

The data synthesis of the analyzed studies will be allocated into the following sections: study characteristics (design, method of randomization (if applicable), blinding, allocation concealment, statistical methods); participants (pregnant and/or postpartum women); interventions (use of sEMG); clinical outcomes (types of outcomes measured: dichotomous or continuous, and adverse effects) (Table 2).

**Table 2:** Study characteristics.

**Authors, Year**	**Pregnancy participants**	**Postpartum participants**	**Gestational age (weeks)**	**EMG protocols**	**Outcomes**	**Conclusions**

Statistical analysis for analysis of subgroup interactions will be analyzed using the Rev-Man tool.

### Sensitivity analysis

A sensitivity analysis will be conducted to obtain a solid conclusion and to evaluate the stability of the results. All analyses will be performed using STATA SE 14.0. The sensitivity analysis may explore the influence of the quality of results. This can be assessed by excluding studies at high risk of bias.

## Discussion

The literature suggests that sEMG, with or without TMAP, can be employed as an effective treatment for pelvic floor dysfunctions, such as SUI. Although clinical trials in the literature provide electromyographic parameters for the treatment of PFMD in pregnant and postpartum women, these data are not summarized. This review aims to analyze the various applied protocols, methods, and terminologies used.

To date, this will be the first systematic review to examine electromyographic patterns in pregnant and postpartum women. These results will investigate whether there is scientific support for clinical practice in the use of electromyography in this population.

## Supporting information

S1 Checklist PRISMA-P (Preferred Reporting Items for Systematic review and Meta Analysis Protocols) 2015 checklist: Recommended items to address in a systematic review protocol. (DOCX)

S1 Appendix (DOCX)

## References

[1] Wu X, Zheng X, Yi X, Lai P, Lan Y. Electromyographic Biofeedback for Stress Urinary Incontinence or Pelvic Floor Dysfunction in Women: A Systematic Review and Meta-Analysis. Adv Ther. 2021 Aug;38(8):4163–4177. doi: 10.1007/s12325-021-01831-6 Epub 2021 Jun 27. ; PMCID: PMC8342347. 34176082 PMC8342347

[2] DeLancey JOL, Masteling M, Pipitone F, LaCross J, Mastrovito S, Ashton-Miller JA. Pelvic floor injury during vaginal birth is life-altering and preventable: what can we do about it? Am J Obstet Gynecol. 2024 Mar;230(3):279–294.e2. doi: 10.1016/j.ajog.2023.11.1253 Epub 2024 Jan 2. ; PMCID: PMC11177602. 38168908 PMC11177602

[3] Falah-Hassani K, Reeves J, Shiri R, Hickling D, McLean L. The pathophysiology of stress urinary incontinence: a systematic review and meta-analysis. Int Urogynecol J. 2021 Mar;32(3):501–552. Epub 2021 Jan 8. Erratum in: Int Urogynecol J. 2021 Jun;32(6):1607. doi: 10.1007/s00192-020-04622-9 ; PMCID: PMC8053188. 33864478 PMC8203550

[4] Woodley SJ, Lawrenson P, Boyle R, Cody JD, Mørkved S, Kernohan A et al. Pelvic floor muscle training for preventing and treatment of urinary and faecal incontinence in antenatal and postnatal women. Cochrane Database Syst Rev. 2020 May 6;5(5):CD007471. doi: 10.1002/14651858.CD007471.pub4 ; PMCID: PMC7203602. 32378735 PMC7203602

[5] He R, Wang X, Nian S, Wang X, Zhang L, Lu Y. The effect of pelvic floor muscle training and perineal massage in late pregnancy on postpartum pelvic floor function in nulliparas: A randomised controlled clinical trial. Complement Ther Med. 2023 Oct;77:102982. doi: 10.1016/j.ctim.2023.102982 Epub 2023 Aug 30. . 37657664

[6] Mateus-Vasconcelos ECL, Ribeiro AM, Antônio FI, Brito LGO, Ferreira CHJ. Physiotherapy methods to facilitate pelvic floor muscle contraction: A systematic review. Physiother Theory Pract. 2018 Jun;34(6):420–432. doi: 10.1080/09593985.2017.1419520 Epub 2017 Dec 26. . 29278967

[7] Dumoulin C, Hay-Smith J. Pelvic floor muscle training versus no treatment, or inactive control treatments, for urinary incontinence in women. Cochrane Database Syst Rev. 2010 Jan 20;(1):CD005654. Update in: Cochrane Database Syst Rev. 2014 May 14;(5):CD005654. doi: 10.1002/14651858.CD005654.pub2 . 20091581

[8] de Oliveira Ferro JK, Lemos A, de Santana Chagas AC, de Moraes AA, de Oliveira-Souza AIS et al. Techniques for Registration of Myoelectric Activity of Women’s Pelvic Floor Muscles: A Scoping Review. Int Urogynecol J. 2024 May;35(5):947–954. doi: 10.1007/s00192-024-05744-0 Epub 2024 Mar 12. . 38472341

[9] Błudnicka M, Piernicka M, Kortas J, Bojar D, Duda-Biernacka B, Szumilewicz A. The influence of one-time biofeedback electromyography session on the firing order in the pelvic floor muscle contraction in pregnant woman-A randomized controlled trial. Front Hum Neurosci. 2022 Sep 29;16:944792. doi: 10.3389/fnhum.2022.944792 ; PMCID: PMC9559232. 36248694 PMC9559232

[10] Marques J, Botelho S, Pereira LC, Lanza AH, Amorim CF, Palma P. et al. Pelvic floor muscle training program increases muscular contractility during first pregnancy and postpartum: electromyographic study. Neurourol Urodyn. 2013 Sep;32(7):998–1003. doi: 10.1002/nau.22346 Epub 2012 Nov 5. . 23129397

[11] Chmielewska D, Stania M, Kucab-Klich K, Błaszczak E, Kwaśna K, Smykla A, et al. Electromyographic characteristics of pelvic floor muscles in women with stress urinary incontinence following sEMG-assisted biofeedback training and Pilates exercises. PLoS One. 2019 Dec 2;14(12):e0225647. doi: 10.1371/journal.pone.0225647 ; PMCID: PMC6886793. 31790463 PMC6886793

[12] Bertotto A, Schvartzman R, Uchôa S, Wender MCO. Effect of electromyographic biofeedback as an add-on to pelvic floor muscle exercises on neuromuscular outcomes and quality of life in postmenopausal women with stress urinary incontinence: A randomized controlled trial. Neurourol Urodyn. 2017 Nov;36(8):2142–2147. doi: 10.1002/nau.23258 Epub 2017 May 16. . 28508398

[13] Nunes EFC, Sampaio LMM, Biasotto-Gonzalez DA, Nagano RCDR, Lucareli PRG, Politti F. Biofeedback for pelvic floor muscle training in women with stress urinary incontinence: a systematic review with meta-analysis. Physiotherapy. 2019 Mar;105(1):10–23. doi: 10.1016/j.physio.2018.07.012 Epub 2018 Sep 24. . 30686479

[14] López-Liria R, Varverde-Martínez MLÁ, Padilla-Góngora D, Rocamora-Pérez P. Effectiveness of Physiotherapy Treatment for Urinary Incontinence in Women: A Systematic Review. J Womens Health (Larchmt). 2019 Apr;28(4):490–501. doi: 10.1089/jwh.2018.7140 Epub 2018 Dec 21. . 30575448

[15] Botelho S, Pereira LC, Marques J, Lanza AH, Amorim CF, Palma P, et al. Is there correlation between electromyography and digital palpation as means of measuring pelvic floor muscle contractility in nulliparous, pregnant, and postpartum women? Neurourol Urodyn. 2013 Jun;32(5):420–3. doi: 10.1002/nau.22321 Epub 2012 Sep 28. . 23023961

[16] Oleksy Ł, Wojciechowska M, Mika A, Antos E, Bylina D, Kielnar R, et al. Normative values for Glazer Protocol in the evaluation of pelvic floor muscle bioelectrical activity. Medicine (Baltimore). 2020 Jan;99(5):e19060. doi: 10.1097/MD.0000000000019060 ; PMCID: PMC7004594. 32000454 PMC7004594

[17] Koenig I, Luginbuehl H, Radlinger L. Reliability of pelvic floor muscle electromyography tested on healthy women and women with pelvic floor muscle dysfunction. Ann Phys Rehabil Med. 2017 Nov;60(6):382–386. doi: 10.1016/j.rehab.2017.04.002 Epub 2017 Jul 14. . 28716538

[18] Koh CE, Young CJ, Young JM, Solomon MJ. Systematic review of randomized controlled trials of the effectiveness of biofeedback for pelvic floor dysfunction. Br J Surg. 2008 Sep;95(9):1079–87. doi: 10.1002/bjs.6303 . 18655219

[19] Fernandes ACNL, Palacios-Ceña D, Pena CC, Duarte TB, de la Ossa AMP, Jorge CH. Conservative non-pharmacological interventions in women with pelvic floor dysfunction: a systematic review of qualitative studies. BMC Womens Health. 2022 Dec 12;22(1):515. doi: 10.1186/s12905-022-02097-y ; PMCID: PMC9743653. 36503437 PMC9743653

[20] Moroni RM, Magnani PS, Haddad JM, Castro Rde A, Brito LG. Conservative Treatment of Stress Urinary Incontinence: A Systematic Review with Meta-analysis of Randomized Controlled Trials. Rev Bras Ginecol Obstet. 2016 Feb;38(2):97–111. doi: 10.1055/s-0035-1571252 Epub 2016 Jan 29. ; PMCID: PMC10309479. 26883864 PMC10309479

[21] Page MJ, McKenzie JE, Bossuyt PM, Boutron I, Hoffmann TC, Mulrow CD, et al. The PRISMA 2020 statement: an updated guideline for reporting systematic reviews. BMJ. 2021 Mar 29;372:n71. doi: 10.1136/bmj.n71 ; PMCID: PMC8005924. 33782057 PMC8005924

[22] Santos CMDC, Pimenta CADM, Nobre MRC. A estratégia PICO para a construção da pergunta de pesquisa e busca de evidências. Rev Lat Am Enfermagem. 2007;15(3):508–1117653438

[23] Carvalho APV, Silva V GA. Avaliação do risco de viés de ensaios clínicos randomizados pela ferramenta da colaboração Cochrane. Diagnóstico Trat [Internet]. 2013;18(1):38–44.

[24] Sterne JAC, Savović J, Page MJ, Elbers RG, Blencowe NS, Boutron I, et al. RoB 2: a revised tool for assessing risk of bias in randomised trials. BMJ. 2019 Aug 28;366:l4898. doi: 10.1136/bmj.l4898 . 31462531

[25] Sterne JA, Hernán MA, Reeves BC, Savović J, Berkman ND, Viswanathan M, et al. ROBINS-I: a tool for assessing risk of bias in non-randomised studies of interventions. BMJ. 2016 Oct 12;355:i4919. doi: 10.1136/bmj.i4919 ; PMCID: PMC5062054. 27733354 PMC5062054

[26] Balshem H, Helfand M, Schünemann HJ, Oxman AD, Kunz R, Brozek J, et al. GRADE guidelines: 3. Rating the quality of evidence. J Clin Epidemiol. 2011 Apr;64(4):401–6. doi: 10.1016/j.jclinepi.2010.07.015 Epub 2011 Jan 5. . 21208779

[27] Leitão A. Electromyographic parameters for treatment of pelvic floor disorders in pregnant and postpartum women: A review protocol. Natl Inst Heal Res. 2023;1–15. 10.1371/journal.pone.0309822 PMC11534233 39495733

