# Supplementary material for: Electromyographic parameters for treatment of pelvic floor disorders in pregnant and postpartum women: A review protocol
Source: PLoS One. 2024 Nov 4;19(11):e0309822. doi: 10.1371/journal.pone.0309822 (PMC11534233; doi:10.1371/journal.pone.0309822)
Supplement: S1 Appendix — (DOCX) [file pone.0309822.s002.docx]

**S1 Appendix**

Search strategy:

**BVS**

(electromyography  OR  "electric myography "  OR  "electrical myography "  OR  "quantitative electromyography "  OR  "electromyographic examination") AND ("pelvic floor disorder"  OR  "pelvic diaphragm disfunction"  OR  "pelvic floor dysfunction"  OR  "pelvic floor disorders") **141 RESULTADOS**

(electromyography  OR  "electric myography "  OR  "electrical myography "  OR  "quantitative electromyography "  OR  "electromyographic examination") AND ("diaphragma pelvis" OR "pelvis floor" OR "pelvic floor disorder"  OR  "pelvic diaphragm disfunction"  OR  "pelvic floor dysfunction"  OR  "pelvic floor disorders") AND ("pregnant woman" OR pregnancy) **10 RESULTADOS**

(electromyography  OR  "electric myography "  OR  "electrical myography "  OR  "quantitative electromyography "  OR  "electromyographic examination") AND ("diaphragma pelvis" OR "pelvis floor" OR "pelvic floor disorder"  OR  "pelvic diaphragm disfunction"  OR  "pelvic floor dysfunction"  OR  "pelvic floor disorders") AND ("postpartum period" OR puerperium OR "post partum period")   **7 RESULTADOS**

(electromyography  OR  "electric myography "  OR  "electrical myography "  OR  "quantitative electromyography "  OR  "electromyographic examination") AND ("diaphragma pelvis" OR "pelvis floor" OR "pelvic floor disorder"  OR  "pelvic diaphragm disfunction"  OR  "pelvic floor dysfunction"  OR  "pelvic floor disorders") AND ("pregnant woman" OR pregnancy) AND ("postpartum period" OR puerperium OR "post partum period")   **4 RESULTADOS**

**Pedro**

pregnant AND Emg AND Pelvic floor

postpartum AND Emg AND Pelvic floor

postpartum AND Biofeedbak AND Pelvic floor

**SCOPUS**

( electromyography  OR  "electric myography "  OR  "electrical myography "  OR  "quantitative electromyography "  OR  "electromyographic examination"  AND  "diaphragma pelvis"  OR  "pelvis floor"  OR  "pelvic floor disorder"  OR  "pelvic diaphragm disfunction"  OR  "pelvic floor dysfunction"  OR  "pelvic floor disorders" )**980 RESULTADOS**

( electromyography  OR  "electric myography "  OR  "electrical myography "  OR  "quantitative electromyography "  OR  "electromyographic examination"  AND  "diaphragma pelvis"  OR  "pelvis floor"  OR  "pelvic floor disorder"  OR  "pelvic diaphragm disfunction"  OR  "pelvic floor dysfunction"  OR  "pelvic floor disorders"  AND  "pregnant woman"  OR  pregnancy  )   **81 RESULTADOS**

 ( electromyography  OR  "electric myography "  OR  "electrical myography "  OR  "quantitative electromyography "  OR  "electromyographic examination"  AND  "diaphragma pelvis"  OR  "pelvis floor"  OR  "pelvic floor disorder"  OR  "pelvic diaphragm disfunction"  OR  "pelvic floor dysfunction"  OR  "pelvic floor disorders"  AND  "postpartum period"  OR  puerperium  OR  "post partum period" ) 39 RESULTADOS

## ( electromyography  OR  "electric myography "  OR  "electrical myography "  OR  "quantitative electromyography "  OR  "electromyographic examination"  AND  "diaphragma pelvis"  OR  "pelvis floor"  OR  "pelvic floor disorder"  OR  "pelvic diaphragm disfunction"  OR  "pelvic floor dysfunction"  OR  "pelvic floor disorders"  AND  "pregnant woman"  OR  pregnancy  AND  "postpartum period"  OR  puerperium  OR  "post partum period" )   27 RESULTADOS
