# Supplementary material for: Electromyographic parameters for treatment of pelvic floor disorders in pregnant and postpartum women: A review protocol
Source: PLoS One. 2024 Nov 4;19(11):e0309822. doi: 10.1371/journal.pone.0309822 (PMC11534233; doi:10.1371/journal.pone.0309822)
Supplement: Attachment — Submitted filename: PONE-D-23-43712_13012024.pdf [file pone.0309822.s003.pdf]

Title	Electromyographic parameters for treating pelvic floor disorders in pregnant and postpartum women: A review protocol
Manuscript No	PONE-D-23-43712

Abstract:

- The abstract needs more detail on the methods - what type of studies will be included, how will they assess risk of bias, etc. Currently it only mentions the databases that will be searched.

- The objective should be more clearly stated - will this review analyze the efficacy of EMG parameters for treating pelvic floor disorders in these populations, or just describe the parameters used? This is unclear.

Introduction:

- The introduction could benefit from more detail on pelvic floor disorders in pregnancy/postpartum and how EMG may help in diagnosis and treatment. Currently it is very brief.

- More rationale is needed on why this review is important - what gap in knowledge is it addressing? How could it improve clinical practice?

Methods:

- The eligibility criteria need significant expansion - what study designs will be included and excluded? What patient population characteristics, types of interventions, comparators, and outcome measures?

- The search strategy requires more detail - search terms, dates searched, and full planned search in each database should be provided.

- Details on the data extraction process, risk of bias assessment, analysis/synthesis, etc. are currently inadequate and require considerable additions following systematic review reporting standards.

- There are no details on evaluation of certainty/quality of evidence. This is an essential component.

Overall, the methods lack sufficient detail at present for a systematic review protocol. There are several key items missing that need to be expanded following PRISMA-P and Cochrane guidelines.

Results:

- This section is underdeveloped since the review has not yet been conducted. But some anticipated analysis/synthesis details could be added.

Discussion:

- The current discussion is very speculative. This should be revised once findings are available to discuss limitations, implications, conclusions, etc.

In summary, while the topic is interesting, the manuscript has substantial gaps in reporting key systematic review methods and requires considerable strengthening of the background, objectives, eligibility criteria, methods, and planned analysis before it could be considered sufficiently rigorous. I would recommend thoroughly revising following systematic review reporting standards. The PROSPERO registration is a good start but significant protocol amendments may be needed before proceeding.
