# Supplementary material for: Electromyographic parameters for treatment of pelvic floor disorders in pregnant and postpartum women: A review protocol
Source: PLoS One. 2024 Nov 4;19(11):e0309822. doi: 10.1371/journal.pone.0309822 (PMC11534233; doi:10.1371/journal.pone.0309822)
Supplement: Attachment — Submitted filename: PONE-D-23-43712.pdf [file pone.0309822.s005.pdf]

PLOS ONE

Electromyographic parameters for treating pelvic floor disorders in pregnant and postpartum women: A review protocol

--Manuscript Draft--

**Manuscript Number:**	PONE-D-23-43712
**Article Type:**	Registered Report Protocol
**Full Title:**	Electromyographic parameters for treating pelvic floor disorders in pregnant and postpartum women: A review protocol
**Short Title:**	Electromyographic parameters for treating pelvic floor disorders: A review protocol
**Corresponding Author:**	ALETHEA CURY RABELO LEITAO, Ms.<br>Federal University of Rio Grande do Norte: Universidade Federal do Rio Grande do Norte<br>Parnamirim, RN BRAZIL
**Keywords:**	Electromyography, pelvic floor disorders, pregnant, postpartum
**Abstract:**	Electromyography is a widely used instrument in clinical practice to evaluate and treat pelvic floor disorders in pregnant and postpartum women.The objective of this study is to analyze the scientific evidence on the electromyography parameters used for treating pelvic floor disorders in pregnant women in any gestational week and postpartum women up to 12 months after delivery.A systematic review will be performed in online databases (Scopus, Medline, Pedro, Scielo and Pubmed) of randomized controlled experimental studies and quasi-experimental studies, in English, Portuguese or Spanish, which used electromyography as an intervention for treating pelvic floor disorders in pregnant or post-childbirth women up to 12 months after birth. Risk of bias assessment will be performed using Cochrane group tools. The Rob 2.0 tool will be used for experimental studies and the Robins-I tool for non-experimental studies. The protocol was registered in PROSPERO (nº.433510). The quality of the evidence will be analyzed using the GRADE System Methodological Guide and the systematic review structure will be performed according to the Preferred Reporting Items for Systematic Reviews and Meta-Analyses (PRISMA) guidelines.
**Order of Authors:**	ALETHEA CURY RABELO LEITAO, Ms.
	Silvia Oliveira Ribeiro Lira, Dra.
	Elizabel de Souza Ramalho Viana, Dra.
**Additional Information:**	
**Question**	**Response**
**Financial Disclosure**<br><br>Enter a financial disclosure statement that describes the sources of funding for the work included in this submission. Review the submission guidelines for detailed requirements. View published research articles from *PLOS ONE* for specific examples.<br><br>This statement is required for submission and **will appear in the published article** if the submission is accepted. Please make sure it is accurate.	NO<br>The funders did not and will not have a role in study design, data collection and analysis, decision to publish, or preparation of the manuscript.

Powered by Editorial Manager® and ProduXion Manager® from Aries Systems Corporation

**Unfunded studies**<br>Enter: *The author(s) received no specific funding for this work.*<br><br>**Funded studies**<br>Enter a statement with the following details:<br>• Initials of the authors who received each award<br>• Grant numbers awarded to each author<br>• The full name of each funder<br>• URL of each funder website<br>• Have or will the sponsors or funders play any role in the study design, data collection (when applicable) and analysis, decision to publish, or preparation of the manuscript?<br>• **NO** - Include this sentence at the end of your statement: *The funders did not and will not have a role in study design, data collection and analysis, decision to publish, or preparation of the manuscript.*<br>• **YES** - Specify the role(s) played.<br><br>* typeset	
**Competing Interests**<br><br>Use the instructions below to enter a competing interest statement for this submission. On behalf of all authors, disclose any competing interests that could be perceived to bias this work—acknowledging all financial support and any other relevant financial or non-financial competing interests.<br><br>This statement is required for submission and **will appear in the published article** if the submission is accepted. Please make sure it is accurate and that any funding sources listed in your Funding Information later in the submission form are also declared in your Financial Disclosure statement.<br><br>View published research articles from *PLOS ONE* for specific examples.	The authors have declared that no competing interests exist.

**NO authors have competing interests**<br><br>Enter: *The authors have declared that no competing interests exist.*<br><br>**Authors with competing interests**<br><br>Enter competing interest details beginning with this statement:<br><br>*I have read the journal's policy and the authors of this manuscript have the following competing interests: [insert competing interests here]*<br><br>* typeset	
**Ethics Statement**<br><br>Enter an ethics statement for this submission. This statement is required if the study involved:<br><br>• Human participants<br>• Human specimens or tissue<br>• Vertebrate animals or cephalopods<br>• Vertebrate embryos or tissues<br>• Field research<br><br>Write "N/A" if the submission does not require an ethics statement.<br><br>General guidance is provided below. Consult the submission guidelines for detailed instructions. **Make sure that all information entered here is included in the Methods section of the manuscript.**	N/A

Powered by Editorial Manager® and ProduXion Manager® from Aries Systems Corporation

**Format for specific study types**<br><br>**Human Subject Research (involving human participants and/or tissue)**<br>• Give the name of the institutional review board or ethics committee that approved the study<br>• Include the approval number and/or a statement indicating approval of this research<br>• Indicate the form of consent obtained (written/oral) or the reason that consent was not obtained (e.g. the data were analyzed anonymously)<br><br>**Animal Research (involving vertebrate animals, embryos or tissues)**<br>• Provide the name of the Institutional Animal Care and Use Committee (IACUC) or other relevant ethics board that reviewed the study protocol, and indicate whether they approved this research or granted a formal waiver of ethical approval<br>• Include an approval number if one was obtained<br>• If the study involved *non-human primates*, add *additional details* about animal welfare and steps taken to ameliorate suffering<br>• If anesthesia, euthanasia, or any kind of animal sacrifice is part of the study, include briefly which substances and/or methods were applied<br><br>**Field Research**<br><br>Include the following details if this study involves the collection of plant, animal, or other materials from a natural setting:<br>• Field permit number<br>• Name of the institution or relevant body that granted permission	
**Data Availability**<br><br>Authors are required to make the data underlying their research findings fully available, without restriction.<br><br>If data need to be collected, modified or processed specifically for your study, or if participants need to be recruited specifically for your study, then it should	Yes - any pilot data reported in this submission are fully available and data collected during the study will be made fully available without restriction upon study completion

occur only after your Registered Report Protocol is accepted for publication.<br><br>If the manuscript reports pilot results, the data underlying those results should be made available at the time of publication. Submission of this Registered Report Protocol implies a commitment that the data collected in the study, upon completion, will be made available with the Registered Report publication.<br><br>When legal or ethical requirements prohibit public data sharing, authors must indicate how researchers may obtain access to the data. See the PLOS Data Policy and FAQ for detailed information.<br><br>A Data Availability Statement describing where the data can be found is required at submission. Your answers to this question constitute the Data Availability Statement and **will be published in the article**, if accepted.<br><br>**Important:** Stating 'data available on request from the author' is not sufficient. If your data are only available upon request, select 'No' for the first question and explain your situation in the text box.<br><br>Do the authors confirm that all data underlying the findings of the study outlined in this Registered Report Protocol will be made fully available without restriction?	
**Describe where the data may be found in full sentences. If you are copying our sample text, replace any instances of XXX with the appropriate details.**<br><br>• If **no pilot data are reported in the manuscript**, please enter the following: *All relevant data from this study will be made available upon study completion.*	All relevant data are within the manuscript and its Supporting Information files

• If the manuscript reports pilot data **held in a public repository**, include URLs, accession numbers or DOIs. If this information will only be available after acceptance, indicate this by ticking the box below. For example: *All XXX files are available from the XXX database (accession number(s) XXX, XXX.).*<br>• If the manuscript reports pilot data contained **within the manuscript and/or Supporting Information files**, enter the following:<br>*All relevant data are within the manuscript and its Supporting Information files.*<br>• If neither of these applies but you are able to provide **details of access elsewhere**, with or without limitations, please do so. For example:<br><br>*Data cannot be shared publicly because of [XXX]. Data are available from the XXX Institutional Data Access / Ethics Committee (contact via XXX) for researchers who meet the criteria for access to confidential data.*<br><br>*The data underlying the results presented in the study are available from (include the name of the third party and contact information or URL).*<br>• This text is appropriate if the data are owned by a third party and authors do not have permission to share the data.<br><br>* typeset	
Additional data availability information:	

Manuscript

Click here to access/download;Manuscript;Manuscript body formatting guidelines.docx

PLOS ONE

Study Protocol Article Template

Electromyographic parameters for treating pelvic floor disorders in pregnant and postpartum women: A review protocol

Alethéa Cury Rabelo Leitão[1#a], Silvia Oliveira Ribeiro Lira[2#b], Elizabel de Souza Ramalho Viana[1#a]

[1] Department of Physical Therapy. Federal University of Rio Grande do Norte, Natal, Brazil

[2] Federal University of Rio Grande do Norte, Natal, Rio Grande do Norte, Faculdade de Ciências da Saúde do Trairi (FACISA/UFRN), Santa Cruz, Rio Grande do Norte, Brazil.

[#a] Current Addres: Department of Physical Therapy. Universidade Federal do Rio Grande do Norte, Natal, Rio Grande do Norte, Brazil

[#b] Current Addres: Federal University of Rio Grande do Norte, Natal, Rio Grande do Norte, Faculdade de Ciências da Saúde do Trairi (FACISA/UFRN), Santa Cruz, Rio Grande do Norte, Brazil.

*Corresponding author
E-mail: ale_cury@yahoo.com.br
These authors contributed equally to this work

Author Contributions

Conceptualization: Alethéa Cury Rabelo Leitão.

Formal analysis: Alethéa Cury Rabelo Leitão, Silvia Oliveira Ribeiro Lira.

Investigation: Alethéa Cury Rabelo Leitão.

Methodology: Alethéa Cury Rabelo Leitão.

Resources: Alethéa Cury Rabelo Leitão, Silvia Oliveira Ribeiro Lira.

Supervision: Elizabel de Souza Ramalho Viana.

Writing – original draft: Alethéa Cury Rabelo Leitão.

Writing – review & editing: Alethéa Cury Rabelo Leitão, Elizabel de Souza Ramalho Viana.

Abstract

Electromyography is a widely used instrument in clinical practice to evaluate and treat pelvic floor disorders in pregnant and postpartum women. The objective of this study is to analyze the scientific evidence on the electromyography parameters used for treating pelvic floor disorders in pregnant women in any gestational week and postpartum women up to 12 months after delivery. A systematic review will be performed in online databases (Scopus, Medline, Pedro, Scielo and Pubmed) of randomized controlled experimental studies and quasi-experimental studies, in English, Portuguese or Spanish, which used electromyography as an intervention for treating pelvic floor disorders in pregnant or post-childbirth women up to 12 months after birth. Risk of bias assessment will be performed using Cochrane group tools. The Rob 2.0 tool will be used for experimental studies and the Robins-I tool for non-experimental studies. The protocol was registered in PROSPERO (nº.433510). The quality of the evidence will be analyzed using the GRADE System Methodological Guide and the systematic review structure will be performed according to the Preferred Reporting Items for Systematic Reviews and Meta-Analyses (PRISMA) guidelines.

Introduction

Description of the condition

It is consensus that the risk factors for developing pelvic floor disorders in women are related to the pregnancy and delivery period[1]. The pelvic floor of these

women will be overloaded during pregnancy due to enlargement of the gravid uterus from anatomical and physiological changes[2]. Therefore, some women may develop pelvic floor disorders (PFD), such as urinary and fecal incontinence and pelvic organ prolapse[3].

Perineal, connective tissue, and muscle rupture or weakness may occur after delivery, which may cause fecal and urinary incontinence, pelvic organ prolapse, sexual dysfunction, and pain syndromes [3]. The literature shows that 50% of women lose some support functionality of the pelvic floor muscles (PFM) due to childbirth, and these injuries increase by an average of 20% in women who had vaginal delivery [4].

Pelvic floor muscle training (PFMT) is the first-line treatment for dysfunctions of this musculature [5]. This treatment is based on increasing strength, endurance, maintaining muscle contraction for a long period of time, muscle coordination, adherence to and motivation for the training program [6]. [7]

Electromyography can be an adjunct to pelvic floor muscle training for treating urinary disorders in pregnant and postpartum women.

Description of the intervention

There are several methods for evaluating and treating the functionality of the pelvic floor muscles, such as: digital palpation, manometry, ultrasound, electromyography and magnetic resonance imaging [6][5][8]

A 2021 meta-analysis with more than four thousand women found that the use of electromyography combined with conservative treatment of the pelvic floor muscles has better results than the isolated treatment[1]

Another 2019 meta-analysis with 1,104 studies did not demonstrate that conservative treatment with electromyographic biofeedback offers a better intervention alternative for the treatment of this urinary incontinence [9]. In other words, it did not find significant differences in the findings when compared with other interventions for the same treatment objective[9][5].

How the intervention will work

Electromyography can be used alone or in combination with conservative treatment. It allows indicating the activity of the pelvic floor muscles in relation to rest during contraction and during relaxation[10][9].

Clinical studies have evaluated the bioelectric activity of this muscle group using electromyography[11]. This resource has been used as an adjunct to conservative training and enables the physiotherapist to correctly and objectively observe the contraction and relaxation of the pelvic floor muscles. Therefore, it facilitates neuromuscular learning and performs rehabilitation more assertively[9][10].

So far, more recent reviews which analyzed the effectiveness of conservative treatment with and without electromyography in relation to pelvic floor strength, urinary incontinence score and quality of sexual life excluded pregnant and postpartum women from the intervention groups to analyze these effects, leaving this gap in the literature on this population[12][13][14].

Knowledge about data on frequency, intensity and type of muscle contraction can determine goals and therapeutic conduct more assertively. The electromyographic parameters of the pelvic floor muscles guide the health professional in choosing the best strategies for the successful treatment of PFM

disorders in pregnant and postpartum women. However, due to the scarcity of studies in this population, a systematic investigation of the literature on the conducts carried out and their effects so far is necessary.

Why is this review important?

It is important to have a better understanding of the electromyography parameters most used in treating pelvic floor muscle disorders in pregnant and postpartum women. Identifying electromyography data can be a reference for elaborating pelvic floor rehabilitation procedures, and thereby contribute to the prevention and treatment of dysfunctions. Furthermore, previous systematic reviews have not analyzed the most appropriate therapy for this population when using EMG alone or in combination in this population.

Materials and Methods

Protocol and guidelines

This review will be conducted following the Preferred Reporting Items for Systematic Reviews and Meta-Analyses (PRISMA) Prisma guidelines[(15)]. The protocol will be registered with PROSPERO and will be carried out between August 2023 and October 2024.

Type of studies

The review will include randomized controlled experimental studies (RCTs) and non-experimental studies (nRCTs), in English, Portuguese or Spanish, using

electromyography in pregnant or postpartum women for evaluating or treating pelvic floor dysfunctions which analyzed baseline tone, contraction and relaxation capacity.

Type of participantes

Studies including pregnant women (at any gestational week) and postpartum women (up to 12 months postpartum) with MAPMD who were treated with EMG will also be considered.

Type of interventions

In addition, studies which used electromyography as an instrument, in isolation or in combination, for evaluating or treating of pelvic floor muscle dysfunctions that describe the parameters used in the therapy of these women will also be included.

Type of outcome mensures

Primary results

1. Baseline pelvic floor muscle tone
2. Maximum voluntary contraction of the pelvic floor muscles
3. Sustained contraction of the pelvic floor muscles
4. Functionality of the pelvic floor muscles (contraction-relaxation coordination capacity)
5. Functionality of the pelvic floor muscles (ability for rapid contractions)

Secondary results

6. Types of electromyographic equipment
7. Patient positioning during the intervention

8. Limitations of the chosen therapy
9. Types of Female Pelvic Floor Dysfunction More
10. Adverse events

Electronic database

1. Cochrane Central Register of Controlled Trials (CENTRAL)
2. MEDLINE (Pubmed)
3. Banco de Dados de Evidências em Fisioterapia (PEDro)
4. Scopus
5. Web of Science
6. Scielo
7. US National Institutes of Health, Register of Continuous Trials, ClinicalTrials.gov (www.clinictrials.gov);

The strategy on the terms used in each database can be found in Appendix S1.

Searching other resources

Reference checking of primary studies will be done manually and review articles will be added to the reference.

Selection of studies

Two independent authors (ACRL and SORL) will analyze the articles by titles and abstracts at the first moment. Eligible studies will be read in full and data extracted for inclusion. The exclusion reasons for the studies will also be analyzed

one by one. Disagreements regarding articles were resolved by a third author by casting vote (ESRV).

Duplicate studies will be identified and excluded. Studies involving men, children or non-pregnant women will also be excluded from the eligibility process and detailed in the Guideline Prisma flowchart.

Data extraction and management

The authors will extract the characteristics below from the included studies:

1. Participants: pregnant women in any gestational phase or postpartum women up to 12 months after delivery, postpartum time, mean age, gestational week, floor dysfunction for treatment, sample inclusion and exclusion criteria, and sample description.
2. Interventi[illegible] type of electromyographic equipment, types of electrodes, type of comparator equipment (if any), use of combined therapy (if any), intervention time, electromyographic parameters used, description of alternative interventions (placebo, no intervention or other intervention).
3. Method: study design, session time, follow-up time, study location, patient positioning, PFM functionality assessment method, treatment method.
4. Results: primary and secondary studies that evaluated electromyographic parameters for treating pelvic floor muscle dysfunctions.
5. Notes: authors, year of publication, funding of studies, and notable conflicts of interest among authors

Two authors (ACRL and SORL) will perform the initial data extraction from the included studies after reading the full text and within the inclusion criteria. A

"Summary of included studies" table will be created, informing the total number of studies. In case of disagreements in the extraction of data, a meeting will first be held for consensus on the extraction, and in the persistence of doubt, a third author will be deciding by the casting vote (ERSV). The review author (ACRL) will transfer the summarized data to the Systematic Reviews management program (RevMan 2014) in order to generate the study report and analysis of heterogeneity and the possibility of meta-analysis of the data.

In case of lack of important data to perform the analysis, one of the review authors will contact yo[illegible]o provide details of the study in question. A professional fluent in the English language or Google Translator will assist in the translation of other published languages in case of doubts. The main results will be carefully reanalyzed by the study authors after translation.

Assessement or risk of bias in the included studies

Two independent authors (ACRL and SORL) will analyze the risk of bias of experimental studies using the Rob 2.0 tool and for non-experimental studies the Robins-I tool. Disagreements will be resolved by consensus or involving a third review author (ESRV).

The Rob 2.0 tool is structured in five domains that have "signaling questions" with the possibility of answers in: "yes", "probably yes", "probably not", "no", "no information'" and "not applicable". Definitive "yes" and "no" answers often indicate that robust evidence is available. The "not applicable" option is only available for questions with a non-mandatory answer. The final score of the responses

determines the risk of bias for each domain: “high risk of bias”, “low risk of bias” or “unclear”[16]

The ROBINS-I tool evaluates seven domains of bias, classified by: low risk of bias, moderate risk of bias, severe risk of bias, critical risk of bias or no information. The result for the final analysis of each component of the domain is based on the answers to the guiding questions and tables that support the judgment of bias in each domain.[17]

Another bias: the ROBINS-I tool also allows for ranking the overall risk of bias, which receives the least favorable ranking among the assessed risks for the assessment tool’s domains.

Evidence quality assessment

The evidence quality will be analyzed using the GRADE tool[18]. The structure of the systematic review will follow the recommendation of the Preferred Reporting Items of Systematic Reviews and Meta-Analyses (PRISMA) guidelines and the protocol is registered in the PROSPERO database, the international prospective register of systematic reviews in health and social care (www.crd.york.ac.uk/prospero).

Assessment of bias during the systematic review

The review must be conducted according to this protocol and any adjustments can be justified in the “Difference between protocol and review” tab in the systematic review session.

Effect treatment measures

Dichotomous data: the odds ratios (ORs) and their associated 95% confidence intervals (CIs) will be used to determine the value of dichotomous data.

Continuous data: will be evaluated using standardized mean differences (STDs) and their corresponding 95% CIs using the Mantel-Haenzel method.

In case of a difference between means, the standard mean can be used for studies that analyzed the same result using different methods. P-values less than 0.05 will be considered statistically significant in all cases.

Issues related to a single analysis

Data analyzed in the study may be analyzed using a single analysis on the outcomes found. Meta-analysis may be used for randomized studies only if the data are justified for doing so.

Lost data

The authors of the present study will be able to contact the authors of the articles listed for data analysis in order to resolve doubts or request numerical data that were not made explicit in the body of the text or which were not found in the Register of Continuous Tests, ClinicalTrials.gov (www.clinictrials.gov). There are cases where only the abstract of the study contains the information, however, in the course of the text it does not. The failure of contacts and loss of entered data cause serious bias and this will be considered in the GRADE system.

Evaluation of heterogeneity

If great heterogeneity is identified between the studies, the possible causes of the data discrepancy can be evaluated using specific sub-groups for analysis. If it is not possible to analyze the subgroup, the qualitative analysis will be summarized and presented in tables.

Evaluation of the risk of bias

The funnel chart can investigate reported biases. Symmetry of the funnel plot can be visually evaluated and explored.

Synthesis of the data

A random effects model and sensitivity analysis with a fixed model can be used for subgroup analysis and data heterogeneity. It is suggested to follow the results below for subgroup analysis:

1. Electromyographic parameters for the treatment of APMD
2. Limitations of the chosen therapy

The following results will be used in subgroup analyzes:

1. Functionality of the pelvic floor muscles
2. Most common types of female pelvic floor dysfunction
3. Adverse events

Statistical analysis for analysis of subgroup interactions will be analyzed using the Rev-Man tool.

Sensitivity analysis

A sensitivity analysis will be conducted to obtain a solid conclusion and to evaluate the stability of the results. All analyses will be performed using STATA SE 14.0. The sensitivity analysis may explore the influence of the quality of results. This can be assessed by excluding studies at high risk of bias.

Discussion

From an evaluation and analysis of the studies that will be systematically reviewed, it is intended to analyze the variation of the protocols tested, methods, terminologies used, as well as definitions of the evaluation of electromyography

components of the pelvic floor and their characteristics. In addition, the analysis of the selected studies on the heterogeneity level of the results is also an objective. The publication of findings may expose gaps in information about the intervention and the standardization of interventions, in addition to comparing results and conclusions between studies.

The publication of this protocol will serve as a reference for elaborating protocols in the rehabilitation of the pelvic floor, identification of the most common dysfunctions, quality of intervention and outcomes, thus contributing to the care and monitoring of these pregnant and postpartum women.

Acknowledgements

None

References

1. Wu X, Zheng X, Yi X, Lai P, Lan Y. Electromyographic Biofeedback for Stress Urinary Incontinence or Pelvic Floor Dysfunction in Women: A Systematic Review and Meta-Analysis. Adv Ther [Internet]. 2021;38(8):4163–77. Available from: https://doi.org/10.1007/s12325-021-01831-6

2. Falah-Hassani K, Reeves J, Shiri R, Hickling D, McLean L. The pathophysiology of stress urinary incontinence: a systematic review and

meta-analysis. Int Urogynecol J. 2021;32(3):501–52.

3. Mørkved S, Bø K. Effect of pelvic floor muscle training during pregnancy and after childbirth on prevention and treatment of urinary incontinence: A systematic review. Br J Sports Med. 2014;48(4):299–310.

4. Caetano AS, Gomes C, Fernandes C, Baena H, Lopes DM. Incontinência urinária e a prática de atividades físicas. Rev Bras Med Esporte. 2007;13(4):270–4.

5. Mateus-Vasconcelos ECL, Ribeiro AM, Antônio FI, Brito LG de O, Ferreira CHJ. Physiotherapy methods to facilitate pelvic floor muscle contraction: A systematic review. Physiother Theory Pract [Internet]. 2018;34(6):420–32. Available from: https://doi.org/10.1080/09593985.2017.1419520

6. Dumoulin C, Cacciari LP, Hay-Smith EJC. Pelvic floor muscle training versus no treatment, or inactive control treatments, for urinary incontinence in women. Cochrane Database Syst Rev. 2018;2018(10):2–186.

7. Paula A, Resende M, Nakamura MU, Alves E, Ferreira G, Petricelli CD, et al. Eletromiografia de superfície para avaliação dos músculos do assoalho pélvico feminino : revisão de literatura. 2011;18(3):292–7.

8. Marques J, Botelho S, LC P, AH L, CF A, Palma P, et al. Pelvic floor muscle training program increases muscular contractility during first pregnancy and postpartum: electromyographic study. [Internet]. Vol. 32, Neurourology and urodynamics. United States; 2013. p. 998–1003. Available from:

https://pubmed.ncbi.nlm.nih.gov/23129397/

9. Nunes EFC, Sampaio LMM, Biasotto-Gonzalez DA, Nagano RC dos R, Lucareli PRG, Politti F. Biofeedback for pelvic floor muscle training in women with stress urinary incontinence: a systematic review with meta-analysis. Physiother (United Kingdom) [Internet]. 2019;105(1):10–23. Available from: https://doi.org/10.1016/j.physio.2018.07.012

10. Koenig I, Luginbuehl H, Radlinger L. Reliability of pelvic floor muscle electromyography tested on healthy women and women with pelvic floor muscle dysfunction. Ann Phys Rehabil Med [Internet]. 2017;60(6):382–6. Available from: http://dx.doi.org/10.1016/j.rehab.2017.04.002

11. Koh CE, Young CJ, Young JM, Solomon MJ. Systematic review of randomized controlled trials of the effectiveness of biofeedback for pelvic floor dysfunction. Br J Surg. 2008;95(9):1079–87.

12. Fernandes ACNL, Palacios-Ceña D, Pena CC, Duarte TB, de la Ossa AMP, Jorge CH. Conservative non-pharmacological interventions in women with pelvic floor dysfunction: a systematic review of qualitative studies. BMC Womens Health [Internet]. 2022;22(1). Available from: https://doi.org/10.1186/s12905-022-02097-y

13. Moroni R, Magnani P, Haddad J, Castro R, Brito L. Conservative Treatment of Stress Urinary Incontinence: A Systematic Review with Meta-analysis of Randomized Controlled Trials. Rev Bras Ginecol e Obs / RBGO Gynecol

Obstet. 2016;38(02):097–111.

14. López-Liria R, Varverde-Martínez MDLÁ, Padilla-Góngora D, Rocamora-Pérez P. Effectiveness of physiotherapy treatment for urinary incontinence in women: A systematic review. J Women's Heal. 2019;28(4):490–501.

15. Page MJ, McKenzie JE, Bossuyt PM, Boutron I, Hoffmann TC, Mulrow CD, et al. The PRISMA 2020 statement: An updated guideline for reporting systematic reviews. BMJ. 2021;372.

16. Sterne JAC, Savović J, Page MJ, Elbers RG, Blencowe NS, Boutron I, et al. RoB 2: A revised tool for assessing risk of bias in randomised trials. BMJ. 2019;366:1–8.

17. Sterne JA, Hernán MA, Reeves BC, Savović J, Berkman ND, Viswanathan M, et al. ROBINS-I: A tool for assessing risk of bias in non-randomised studies of interventions. BMJ. 2016;355:4–10.

18. Balshem H, Helfand M, Schünemann HJ, Oxman AD, Kunz R, Brozek J, et al. GRADE guidelines: 3. Rating the quality of evidence. J Clin Epidemiol. 2011;64(4):401–6.

Supporting information

S1 Checklist. PRISMA-P (Preferred Reporting Items for Systematic review and Meta

Analysis Protocols) 2015 checklist: Recommended items to address in a systematic review protocol.(DOC)

S1 Appendix.(DOCX)

Prisma - P

Click here to access/download

Supporting Information

3. Supporting Information (Prisma-P).docx

Click here to access/download
Supporting Information
4. S1 Appendix.docx
