# Supplementary material for: Electromyographic parameters for treatment of pelvic floor disorders in pregnant and postpartum women: A review protocol
Source: PLoS One. 2024 Nov 4;19(11):e0309822. doi: 10.1371/journal.pone.0309822 (PMC11534233; doi:10.1371/journal.pone.0309822)
Supplement: Attachment — Submitted filename: PLUS ONE. March.docx [file pone.0309822.s004.docx]

This study surveys Electromyographic parameters for treating pelvic floor disorders in pregnant and postpartum women: A review protocol.

It is an interesting and new topic. The comments below may help you rewrite the article.

- In the title: “Electromyographic parameters for treating pelvic floor disorders in pregnant........”, do you want to survey EMG parameters or treatment effects?

- Please determine whether you want to include studies that used electromyography as an intervention or assessment. These two types of studies will be different from each other. You wrote intervention somewhere in the manuscript and evaluation somewhere else. Please clarify this issue. It would be better to exclude studies that only evaluated electromyography and include studies that used EMG biofeedback as a treatment.

- Which type of pelvic floor disorders would you like to include? Urinary (incontinence, retention...), bowel (constipation, incontinence…), or sexual disorders? These are different from each other and have different treatment protocols. I think it is better to limit the inclusion criteria.

- What do MAPMD in line 131 and APMD in line 269 stand for?
