# Supplementary material for: Electromyographic parameters for treatment of pelvic floor disorders in pregnant and postpartum women: A review protocol
Source: PLoS One. 2024 Nov 4;19(11):e0309822. doi: 10.1371/journal.pone.0309822 (PMC11534233; doi:10.1371/journal.pone.0309822)
Supplement: Attachment — Submitted filename: Response to reviewers.docx [file pone.0309822.s006.docx]

**RESPONSE TO REVIEWERS**

**REVIEW 1**

**ABSTRACT**

**Question 1:** The abstract needs more detail on the methods - what type of studies will be included, how will they assess risk of bias, etc. Currently it only mentions the databases that will be searched.

**Response**

The section was rewritten detailed the types of studies to be included, the assessment of risk of bias using the Cochrane tool and the mention of the study's databases.

**Question 2:** The objective should be more clearly stated - will this review analyze the efficacy of EMG parameters for treating pelvic floor disorders in these populations, or just describe the parameters used? This is unclear.

**Response**

The text was rewritten to emphasize the main objective of the research: the treatment of pelvic floor dysfunctions using sEMG (p. 6). Sections mentioning pelvic floor assessment were removed. In the “Type of studies” and “Type of interventions” sections RCTs and non-RCTs using sEMG as a treatment were specified (p. 7).

**INTRODUCTION**

**Question 1:** The introduction could benefit from more detail on pelvic floor disorders in pregnancy/postpartum and how EMG may help in diagnosis and treatment. Currently it is very brief.

**Response**

The introduction has been rewritten following models from previous publications in the journal PLOS ONE (Barbosa et al., 2020^[1]^; Lira et al., 2022^[2]^ ; Zielinski et al., 2023^[3]^). Additional information has been included on the epidemiology of dysfunctions, the effects of pathologies on women's health, the different presentations of dysfunctions and electromyography treatment in pregnant women who have had vaginal deliveries. In the end, the importance of the study has been more thoroughly detailed(p.3-7).

**Question 2:** More rationale is needed on why this review is important - what gap in knowledge is it addressing? How could it improve clinical practice?

**Response**

The section was rewritten to address the issue comprehensively. The role of sEMG in treating common dysfunctions within the study population such as stress urinary incontinence and pudendal nerve injury during vaginal delivery was highlighted. The importance of the current review as a contribution to defining parameters used in the treatment of pelvic floor dysfunctions in pregnant and postpartum women was emphasized(p.5-6).

**METHODS**

**Question 1:** The eligibility criteria need significant expansion - what study designs will be included and excluded? What patient population characteristics, types of interventions, comparators, and outcome measures?

**Response**

The steps for data extraction and management were detailed based on the PICO acronym. Each letter was selected to better formulate the research question and guide the reader through the steps for the search strategy of eligible articles necessary for data extraction. The following table was added at the end (p.8).

PICO:P (Population): Specifies the population or patient group of interest, I (Intervention): Defines the intervention being studied, C (Comparison): Identifies the comparison group or treatment, O (Outcome): Describes the outcomes of interest.

**Question 2**: The search strategy requires more detail - search terms, dates searched, and full planned search in each database should be provided.

**Response**

The search strategy was included in S1 Appendix, highlighting the descriptors to be used and the Boolean operators with their combinations. (electromyography OR "electric myography " OR "electrical myography " OR "quantitative electromyography " OR "electromyographic examination") AND ("pelvic floor disorder" OR "pelvic diaphragm disfunction" OR "pelvic floor dysfunction" OR "pelvic floor disorders")

**Question 3**: Details on the data extraction process, risk of bias assessment, analysis/synthesis, etc. are currently inadequate and require considerable additions following systematic review reporting standards.

**Response**

The assessment of bias risk in experimental and quasi-experimental studies will be conducted according to the Cochrane Handbook of Intervention Systematic Reviews. The selection of these study designs was based on the rationale that they provide therapeutic intervention data for a specific sample. For this research, the Rob 2.0 tools will be used for experimental studies, and Robins-I for non-experimental studies(p.9)

**Question 4:** There are no details on evaluation of certainty/quality of evidence. This is an essential component.

**Response**

The evidence quality will be analyzed using the GRADE tool. The structure of the systematic review will follow the recommendation of the Preferred Reporting Items of Systematic Reviews and Meta-Analyses (PRISMA) guidelines and the protocol is registered in the PROSPERO database , the international prospective register of systematic reviews in health and social care ([www.crd.york.ac.uk/prospero](http://www.crd.york.ac.uk/prospero)) (p.10).

**Question 5:** Overall, the methods lack sufficient detail at present for a systematic review protocol. There are several key items missing that need to be expanded following PRISMA-P and Cochrane guidelines.

**Response**

The section was rewritten to justify the selection of the study types, and consequently, the tools for assessing the risk of bias in the included studies were detailed. Regarding other risks of bias and the synthesis of information (p.12).

**RESULTS**

**Question 1:** This section is underdeveloped since the review has not yet been conducted. But some anticipated analysis/synthesis details could be added.

**Response**

The sectrion will find in S1File. We anallysed PRISMA-P (Preferred Reporting Items for Systematic review and Meta-Analysis Protocols) 2015 check list: Recommended items to address in a systematic review protocol)

**DISCUSSION**

**Question 1:** The current discussion is very speculative. This should be revised once findings are available to discuss limitations, implications, conclusions, etc.

**Response**

The discussion section was rewritten to emphasize the gap in the scientific literature on the topic, the clinical implications, and the importance of the review's results for professionals using surface electromyography in their clinical practice (p.12).

**REVIEW 2**

**Question 1:** In the title: “Electromyographic parameters for treating pelvic floor disorders in pregnant........”, do you want to survey EMG parameters or treatment effects?

**Response**

The research aims to analyze the electromyographic parameters used in the treatment of pelvic floor dysfunctions.

**Question 2:** Please determine whether you want to include studies that used electromyography as an intervention or assessment. These two types of studies will be different from each other. You wrote intervention somewhere in the manuscript and evaluation somewhere else. Please clarify this issue. It would be better to exclude studies that only evaluated electromyography and include studies that used EMG biofeedback as a treatment.

**Response**

The text was rewritten to emphasize the main objective of the research: the treatment of pelvic floor dysfunctions using sEMG (p. 6). Sections mentioning pelvic floor assessment were removed. In the “Type of studies” and “Type of interventions” sections, RCTs and non-RCTs using sEMG as a treatment were specified (p. 7).

**Question 3:** Which type of pelvic floor disorders would you like to include? Urinary (incontinence, retention...), bowel (constipation, incontinence…), or sexual disorders? **Response**

These are different from each other and have different treatment protocols. I think it is better to limit the inclusion criteria.

**Question 4:** Where was it written “electromyography in pregnant or postpartum women for evaluation or treating pelvic floor dysfunctions (IUE) which analyzed baseline tone, contraction and relaxation capacity.”

**Response**

It has been corrected to “sEMG in pregnant or postpartum women for treating pelvic floor dysfunctions (IUE) which analyzed baseline tone, contraction and relaxation capacity.”

**Question 5:** What do MAPMD in line 131 and APMD in line 269 stand for?

**Response**

MAPMD is the acronym for "disfunção dos músculos do assoalho pélvico" in the native language (Portuguese). The term has been translated to English as "pelvic floor muscle dysfunction (PFMD)." Where was it written MAPMD, it has been corrected to PFMD.

**REVIEW 3**

**DESCRIPTION OF THE CONDITION**

**Question 1:** Line 57: Please follow usual approaches to writing an introduction. Consider reading published articles from PlosONE to see how their introductions are written

**Question 2:** Line 63: This is a good attempt but its largely descriptive; which may be fine. However, you need to give your reader an understanding of the burden (whether globally or nationally) to warrant further review or exploration of the evidence. Also, your subheading reads 'description of condition', however, you are also describing PFMT. Is this standard practice? Consider avoiding subheading or if you decide to use them, stay within the remit of the subheading. Either way, please make your introduction more coherent to build a case for the review.

**Response to line 57 e 63**

The introduction has been rewritten following models from previous publications in the journal PLOS ONE (Barbosa et al., 2020^[1]^; Lira et al., 2022^[2]^ ; Zielinski et al., 2023^[3]^). Additional information has been included on the epidemiology of dysfunctions, the effects of pathologies on women's health, the different presentations of dysfunctions and electromyography treatment in pregnant women who have had vaginal deliveries. In the end, the importance of the study has been more thoroughly detailed(p.3-7).

.

**DESCRIPTION OF THE INTERVENTION**

**Question 1:** Line 76: The rationale for this section is not clear. Are you trying to identify a gap within the evidence to make a case for your review? If so you need to be explicit about the gaps and the start to make a case for further studies. Again, this section did not describe an intervention but only reported previous reviews that have applied certain interventions. Those interventions need to be described based on your subheading.

**Response:**

The justification for the study has been rewritten with more detail. In the first two paragraphs, the description of the intervention begins by defining surface electromyography (sEMG), its mode of use and a description of the intervention. The gaps in the literature have been better described through studies on different findings. Finally, the data that are important to investigate in the treatment using EMG in pregnant and postpartum women have been presented.

**Question 2:** Line 83: It will be prudent to report the number of studies that were included in the meta-analysis rather than the studies that were retrieved on initial search.

**Response**

The meta-analysis by Nunes et al. (2019) was described in greater detail (11 studies). A new systematic review by Chmielewska et al. (2019), including a sample of 12 studies, was added to the text.

**Question 3:** Line 86: What were the effect sixes and p-values?

**Response**

The systematic review by Mateus-Vasconcelos et al. (2018) was detailed further regarding the characteristics analyzed by electromyography, which can be investigated in the scientific research. The meta-analysis by Nunes et al. (2019) was better described in the text, highlighting the use of BFB-EMG in the training of pelvic floor muscles in women with SUI. Consequently, the effects of the study objectives were detailed, and the current topic's objective was discussed. The studies show a p-value < 0.05

**HOW THE INTERVENTION WILL WORK**

**Question 1:** Line 88: Are you making the argument that studies do not report optimal parameters for electromyography? If so, how do you determine that there should be an optimal dosage? Also, what is the significance of EMG since it is not the treatment but a means of monitoring? Will the monitoring with EMG change the parameters regarding conservative treatment? If not what will be the value of knowing the optimal parameters for the EMG. Is it the case that there are optimal parameters for conservative management for pelvic floor disorders and the can be used to monitor the muscle activity and improvement? You just need to be clear regarding what the value of knowing the parameters of EMG for conservative management of pelvic floor disorders.

**Response**

The section was rewritten with more details about EMG and its characteristics. Initially, electromyography was defined and its utility in aiding healthcare professionals in clinical practice was explained. Additionally, the characteristics important for decision-making in the treatment of pelvic floor dysfunctions in pregnant and postpartum women were described. This study will contribute to the production of more scientific evidence on the subject.

**WHY IS THIS REVIEW IMPORTANT?**

**Question 1:** Line 116: My understanding is that EMG monitors muscular activity to guide diagnosis of muscular disorders. In that sense, it can also guide or demonstrates muscles that are improving with management. I wonder what an optimal parameter will mean in terms of a muscular disorder? What grade of injury to the muscle will you consider? You need a strong justification for your review.

**Response**

The section was rewritten to address the issue comprehensively. The role of sEMG in treating common dysfunctions within the study population such as stress urinary incontinence and pudendal nerve injury during vaginal delivery was highlighted. The importance of the current review as a contribution to defining parameters used in the treatment of pelvic floor dysfunctions in pregnant and postpartum women was emphasized.

**TYPE OF STUDIES**

**Question 1:** Line 127: Will EMG be used in treatment or monitor improvement as a result of treatment?

**Response**

Where was it written “electromyography in pregnant or postpartum women for evaluation or treating pelvic floor dysfunctions (IUE) which analyzed baseline tone, contraction and relaxation capacity.”, it has been corrected to “sEMG in pregnant or postpartum women for treating pelvic floor dysfunctions (IUE) which analyzed baseline tone, contraction and relaxation capacity.”

**TYPE OF PARTICIPANTES**

**Question 1**: Line 129: Check your spellings

**Response**

Where was it written “including.”, it has been corrected to “will be included.”

**Question 2:** Line 131: O que é a MAPMD? Já o apresentou e explicou?

**Response**

MAPMD is the acronym for "disfunção dos músculos do assoalho pélvico" in the native language (Portuguese). The term has been translated to English as "pelvic floor muscle dysfunction (PFMD)."Where was it written MAPMD, it has been corrected to PFMD.

**SELECTION OF STUDIES**

**Question 1:** Line 171: Please check you tenses

**Response**

Where was it written “Disagreements regarding articles were resolved by a third author by casting vote (ESRV)”, it has been corrected to disagreements regarding articles will be resolved by a third author by casting vote (ESRV)

**DATA EXTRACTION AND MANAGEMENT**

**Question 1:** Line 176: How did you determine this data extraction procedure? Is it based on evidence? Are there evidence-based data extraction tools you can apply?

**Response**

The steps for data extraction and management were detailed based on the PICO acronym. Each letter was selected to better formulate the research question and guide the reader through the steps for the search strategy of eligible articles necessary for data extraction. The following table was added at the end (p.8).

PICO:P (Population): Specifies the population or patient group of interest, I (Intervention): Defines the intervention being studied, C (Comparison): Identifies the comparison group or treatment, O (Outcome): Describes the outcomes of interest.

**Question 2**: Line 182: Is EMG an intervention or a diagnostic tool?

**Response**

The entire section on the characteristics of the included studies has been reorganized. The detailed PICO strategy highlights EMG as the intervention for treating PFMD.

**Question 3:** Line 202: Who is you?

**Response**

I am the reviewer responsible for searching for new articles (ACRL).

**ASSESSEMENT OR RISK OF BIAS IN THE INCLUDED STUDIES**

**Question 1:** Line 208: You need to justify the basis for considering experimental and non-experimental studies. Which of your study designs target non-experimental studies? This is unclear. How do you plan to use different tools and synthesize the information.

**Response**

The section was rewritten to justify the selection of the study types, and consequently, the tools for assessing the risk of bias in the included studies were detailed. Regarding other risks of bias and the synthesis of information.

**EVALUATION OF HETEROGENEITY**

**Question 1:** Line 260: How can you perform a qualitative analysis of quantitative data? This is unclear.

**Response**

This analysis will be obtained using the Cochrane's Q test, which includes the I2 statistic and the P-value obtained in the test.

**EVALUATION OF THE RISK OF BIAS**

**Question 1:** Line 263 - How will this process be appropriate for non-experimental studies?

**Response**

The assessment of publication bias will be analyzed using the funnel plot and the Egger's test.

**SYNTHESIS OF THE DATA**

**Question 1**: Line 274 - How will this process be applied to non-experimental studies?

**Response**

The synthesis of data from the analyzed studies will be allocated into the following sections: study characteristics (design, method of randomization (if applicable), blinding, allocation methods, statistical analysis); participants (pregnant and/or postpartum women); interventions (use of sEMG); clinical outcomes (types of outcomes measured: dichotomous or continuous, and adverse effects).

**DISCUSSION**

**Question 1**: Line 294 **-** I am not convinced about the need for this review. If you are using EMG as a diagnostic tool and also guide conservative management, you need to make it clear what the value of knowing the parameters on the EMG per patient. Will this be used as a target parameter for management? Will that be possible to determine given that various factors may contribute to recovery, peoples' body constitution may impact readings, and level of muscle disorder can contribute to parameter determination. Please consider these and revise the protocol accordingly. This is a very good attempt.

**Response**

The discussion section was rewritten to emphasize the gap in the scientific literature on the topic, the clinical implications, and the importance of the review's results for professionals using surface electromyography in their clinical practice (p.12).
