# Supplementary material for: Electromyographic parameters for treatment of pelvic floor disorders in pregnant and postpartum women: A review protocol
Source: PLoS One. 2024 Nov 4;19(11):e0309822. doi: 10.1371/journal.pone.0309822 (PMC11534233; doi:10.1371/journal.pone.0309822)
Supplement: Attachment — Submitted filename: 2. Response to reviewrs.docx [file pone.0309822.s007.docx]

**RESPONSE TO REVIEWERS**

Dear Reviewers:

We hope this email finds you well.

We would like to express our gratitude once again for the opportunity to revise our work, and we hope that the new version of the manuscript meets the expectations of the journal.

Below, we highlight the changes made in response to considerations. We remain available for any further clarifications that may be required.

Best Regards,

Alethéa Cury Rabelo Leitão

Federal University of Rio Grande do Norte

Department of Physical Therapy

**JOURNAL REQUIREMENTS:**

Question:

Please review your reference list to ensure that it is complete and correct. If you have cited papers that have been retracted, please include the rationale for doing so in the manuscript text, or remove these references and replace them with relevant current references. Any changes to the reference list should be mentioned in the rebuttal letter that accompanies your revised manuscript. If you need to cite a retracted article, indicate the article’s retracted status in the References list and and also include a citation and full reference for the retraction notice.

Response

We have replaced references 2, 4, 8, 9, and 15. All references have been updated without compromising the scientific content.

**Reference 2:**
Before:
Caetano AS, Gomes C, Fernandes C, Baena H, Lopes DM. Incontinência urinária e a prática de atividades físicas. Rev Bras Med Esporte. 2007;13(4):270–4.

After:
DeLancey JOL, Masteling M, Pipitone F, LaCross J, Mastrovito S, Ashton-Miller JA. Pelvic floor injury during vaginal birth is life-altering and preventable: what can we do about it? Am J Obstet Gynecol. 2024 Mar;230(3):279-294.e2. doi: 10.1016/j.ajog.2023.11.1253. Epub 2024 Jan 2. PMID: 38168908; PMCID: PMC11177602.

**Reference 4:**
Before:
Mørkved S, Bø K. Effect of pelvic floor muscle training during pregnancy and after childbirth on prevention and treatment of urinary incontinence: A systematic review. Br J Sports Med. 2014;48(4):299–310.

After:
Woodley SJ, Lawrenson P, Boyle R, Cody JD, Mørkved S, Kernohan A, Hay-Smith EJC. Pelvic floor muscle training for preventing and treating urinary and faecal incontinence in antenatal and postnatal women. Cochrane Database Syst Rev. 2020 May 6;5(5):CD007471. doi: 10.1002/14651858.CD007471.pub4. PMID: 32378735;

**Reference 8:**
Before:
Paula A, Resende M, Nakamura MU, Alves E, Ferreira G, Petricelli CD, et al. Eletromiografia de superfície para avaliação dos músculos do assoalho pélvico feminino: revisão de literatura. 2011;18(3):292–7.

After:
de Oliveira Ferro JK, Lemos A, de Santana Chagas AC, de Moraes AA, de Oliveira-Souza AIS, de Oliveira DA. Techniques for Registration of Myoelectric Activity of Women's Pelvic Floor Muscles: A Scoping Review. Int Urogynecol J. 2024 May;35(5):947-954. doi: 10.1007/s00192-024-05744-0. Epub 2024 Mar 12. PMID: 38472341.

**Reference 9:**
Before:
Batista RLA, Franco MM, Naldoni LM V., Duarte G, Oliveira AS, Ferreira CHJ. Biofeedback and the electromyographic activity of pelvic floor muscles in pregnant women. Brazilian J Phys Ther [Internet]. 2011 Oct [cited 2015 Jun 13];15(5):386–92.

After:
Błudnicka M, Piernicka M, Kortas J, Bojar D, Duda-Biernacka B, Szumilewicz A. The influence of one-time biofeedback electromyography session on the firing order in the pelvic floor muscle contraction in pregnant woman-A randomized controlled trial. Front Hum Neurosci. 2022 Sep 29;16:944792. doi: 10.3389/fnhum.2022.944792. PMID: 36248694;.

**Reference 15:**
Before:
Marques J, Botelho S, Pereira LC, Lanza AH, Amorim CF, Palma P, Riccetto C. Pelvic floor muscle training program increases muscular contractility during first pregnancy and postpartum: electromyographic study. Neurourol Urodyn. 2013 Sep;32(7):998-1003. doi: 10.1002/nau.22346. Epub 2012 Nov 5. PMID: 23129397.

After:
Botelho S, Pereira LC, Marques J, Lanza AH, Amorim CF, Palma P, Riccetto C. Is there correlation between electromyography and digital palpation as means of measuring pelvic floor muscle contractility in nulliparous, pregnant, and postpartum women? Neurourol Urodyn. 2013 Jun;32(5):420-3. doi: 10.1002/nau.22321. Epub 2012 Sep 28. PMID: 23023961.

.

**REVIEW 2**

**Question:**
Most of the requested items have been modified and implemented. Question 3 has not been answered. It is better for the authors to pay attention to this issue or to use sub-group analysis for each type of pelvic floor dysfunction.

**Response:**
Before: "Electromyographic parameters for the treatment of APMD"
After: "Electromyographic parameters for the treatment of stress urinary incontinence in pregnancy and postpartum women."

The term "APMD" was replaced with "stress urinary incontinence in pregnancy and postpartum women" to clarify the specific dysfunction being investigated in this protocol.

**REVIEW 4**

**Question:**
All comments have been addressed; however, an English language revision is needed.

**Response:**
The English language revision has been completed
